# Transformative interprofessional education and campus community partnerships to prepare the health professions workforce for advancing health equity

**DOI:** 10.3389/fpubh.2026.1763427

**Published:** 2026-03-25

**Authors:** Memoona Hasnain, Valerie Gruss, Michael J. Koronkowski, Sonia Oyola, Susan M. Walsh, Steven Andes, Khatija Noorullah, Kanwal Haque

**Affiliations:** 1 Department of Family and Community Medicine, College of Medicine, University of Illinois Chicago, Chicago, IL, United States; 2 BioBehavioral Nursing Science, College of Nursing, University of Illinois Chicago, Chicago, IL, United States; 3 College of Pharmacy, University of Illinois Chicago, Chicago, IL, United States; 4 Department of Family Medicine, University of Chicago, Chicago, IL, United States; 5 Human Development Nursing Science, College of Nursing, University of Illinois Chicago, Chicago, IL, United States; 6 School of Public Health, University of Illinois Chicago, Chicago, IL, United States; 7 Department of Population Oral Health, College of Dentistry, University of Illinois Chicago, Chicago, IL, United States

**Keywords:** campus community partnerships, CBPR (community-based participatory research), health equity, interprofessional education, interprofessional education and collaborative practice

## Abstract

**Introduction:**

This paper presents work in the continuum of campus-community partnerships to train health science students to advance health equity. The authors share the development, implementation, and evaluation of “Interprofessional Approaches to Health Disparities” (IAHD), a course offered at the University of Illinois Chicago, a large, urban, public, academic institution.

**Methods:**

The IAHD course focuses on selected vulnerable populations: Geriatrics, HIV/AIDS, Homelessness, Immigrant and Refugee Health, Incarcerated Populations, and Intimate Partner Violence. Health science students from medicine, nursing, pharmacy, dentistry, and public health learn in interprofessional teams, with didactic and experiential learning activities. Training includes addressing social determinants of health through interprofessional education and mentored community-based participatory research projects. The Kirkpatrick’s framework was used for program evaluation, with a retrospective pre-post design to measure self-efficacy for competence in interprofessional collaborative practice (IPC) using IPECC-SET, a validated instrument. The evaluation goal was to explore the impact of the learning experiences on attainment of learning objectives. Descriptive analysis of quantitative data explored the extent to which the learning objectives were met. Paired t-tests examined pre-post differences in self-efficacy for competence in IPC. Thematic analysis was conducted for open-ended qualitative data.

**Results:**

Since its inception, 202 students have participated in the course. Program evaluation results indicate that students view the learning experiences positively; cumulatively, over 90% agreed that the course learning objectives were met. Analysis of the IPECC-SET data demonstrated statistically significant (*p* < 0.001) increases in self-efficacy for IPC across all 38 items, in all four domains. Qualitative data analysis yielded themes regarding the most effective aspects of the course and how learning experiences prepared students for their future work.

**Discussion:**

Thus far, eleven cohorts of interprofessional health science students have been trained as a cadre of future health professionals, encouraged to continue practicing advocacy, leadership, scholarship, and interprofessional teamwork to advance health equity. The IAHD course is an exemplar of “*Education in Action*” and has led to meaningful contributions to the training of the future health professions workforce. It addresses workforce development needs, with a special focus on mitigating health disparities and advancing health equity to meet the challenges of the 21st century.

## Introduction

*“Of all the forms of inequality, injustice in health is the most shocking and the most inhuman because it often results in physical death.”—*Dr. Martin Luther King Jr.

This paper describes the work at the University of Illinois Chicago (UIC), a large urban public university, in developing, implementing, and evaluating transformative interprofessional education in the form of a course titled “Interprofessional Approaches to Health Disparities” (IAHD). The IAHD course is designed to prepare health professions students to become future leaders, scholars, advocates, and change agents advancing health equity. We share lessons learned from our journey in the continuum of campus-community partnerships, focusing on training health science students in community-based participatory research (CBPR) as the foundational approach to addressing health inequities among selected vulnerable populations. Our work is grounded in addressing social determinants of health and building and sustaining campus-community partnerships with Chicago Area Community Agencies serving six extremely vulnerable populations: older adults, persons living with HIV/AIDS, the homeless, immigrants and refugees, incarcerated populations, and survivors of intimate partner violence.

Despite growing recognition of the quality gaps in health care access and delivery in the United States, health inequities persist. Evidence from leading national agencies, such as the National Institutes of Health, the Institute of Medicine (IOM), and the Centers for Disease Control and Prevention, suggests that health care inequities persist across diverse populations (1–5). Racial and ethnic minorities have less access to care and are less likely to receive needed routine medical procedures. They experience lower-quality health care, even when their age, medical condition severity, income, and insurance status are comparable. These inequities exist at both national and local levels, and the reasons for these inequities are complex, with social factors disproportionately responsible for inequities in health outcomes, and systemic racism being a root cause (6). Policies shaping social determinants and public health agendas often contribute to higher rates of morbidity and mortality among people of color (7, 8).

Addressing health inequities requires healthcare reform, redesign, the development of new models of healthcare delivery, and the thoughtful integration of transformative models of healthcare professions education. Key recommendations from a seminal report, *Revisiting the Medical School Mission at a Time for Expansion* (9) by the Josiah Macy Junior Foundation include: (1) acceleration in the pace of change in order to prepare future physicians to meet the public’s increasingly demanding needs and expectations; (2) medical educators to ensure that physicians have more training in population health and the role social factors play in effecting health change; and (3) more frequent use of community-based settings as learning environments and less frequent use of hospital-based settings.

Regarding medicine, the calls to transform medical education to include interprofessional education led to the Liaison Committee on Medical Education Accreditation Standard ED-19-A, which stipulates that the core curriculum of a medical education program must prepare students to function collaboratively on healthcare teams. This standard aims to ensure graduates possess the necessary skills to work effectively with other health professionals to provide high-quality patient care, leading to improved patient outcomes, enhanced safety, and quality of care (10). Similar calls to transform health professions education have also come from other fields, including nursing, dentistry, pharmacy, and public health, the participating professions in the IAHD course. Nursing reforms for education emphasize competency-based preparation and team-based care, with national standards identifying interprofessional partnerships as a core component of professional nursing practice and health system improvement (11, 12). Dentistry is prioritizing integrating oral health into team-based care, recognizing that interprofessional education helps reduce disciplinary silos and supports more comprehensive, patient-centered health care (13). Pharmacy education similarly supports interprofessional training to prepare students for collaborative, coordinated care delivery, with professional organizations highlighting teamwork as essential to improving medication management and patient outcomes (14, 15). Public Health also highlights interprofessional education as critical for addressing complex population health challenges, promoting coordinated services, and strengthening collaboration across clinical and community systems (16). Traditional health professions education often occurs in silos, with limited opportunities for doctors, nurses, pharmacists, and other healthcare professionals to learn and practice in interprofessional teams during their formative years. A wealth of evidence supports the effectiveness of interprofessional training in delivering high-quality healthcare, fostering collaborative relationships among healthcare professionals and communities, and ultimately facilitating the provision of patient-centered care, a cornerstone of quality, as designated by the IOM, and advancing health equity (17–23).

Chicago, where the IAHD course is offered, is the third most populous city in the United States, with a long history as a gateway for immigrants. Between the 1940s and the 1970s, five million African Americans made the Great Migration from the rural South to the North. Chicago neighborhood demographics have evolved to a mix of White (31.4%), African American (28.7%), Hispanic/Latino (29.6%), and Asian (6.9%). As of 2023, 20.6% of Chicago, IL residents were born outside of the country (557 k people) (24). There is strong and persistent evidence of health inequities in the Chicago metropolitan area. Alarmingly, some health inequities, particularly among certain minority populations and involving specific health-related issues, are more pronounced at the local level than the national level. Chicago has substantial numbers of underserved communities with documented healthcare needs and inadequate access to healthcare services (25–27). The growing number of underserved and vulnerable populations locally and nationally mandates that these pervasive issues in health and society be addressed. To prepare the future health professions workforce to address health inequities and care for vulnerable populations, we identified critical areas of unmet need that must be integrated into health professions education. The following six concentrations were selected because they represent at-risk populations with unique and unprecedented demands for essential healthcare services, require extensive public health resources and support, and demonstrate a tremendous impact on an ill-equipped healthcare system to address their health and well-being.

### Geriatrics

The growing number of older adults in the U.S. population has placed unprecedented demands on aging services, public health, and health care systems. The U.S. older adult population grew from 40 million in 2020 to 61.2 million in 2024. By 2034, one in five Americans will be over 65 years old, and there will be more older adults than children (28), significantly changing the U.S. demographics. In Chicago, the most recent demographic estimates from the US Census Bureau’s American Community Survey (2019–2023) indicate that adults 65 years and older make up about 13.3% of the city’s population (29). A 2025 Senior Needs Assessment for Chicago highlights the ongoing socioeconomic and health challenges among older adults, showing that senior poverty, seniors living alone, food insecurity, and disability rates have either increased or remained stable, indicating persistent needs for planning for health and social services (29). These issues are compounded by an insufficient health workforce. As the US population ages, the supply of clinicians trained in geriatrics has not kept pace, creating a workforce gap that is expected to widen over time (30). Including geriatrics as a concentration in our training aligns with guidance from the IOM report, Retooling for an Aging America (31), which recommends that training be geared toward providing geriatrics education to all clinicians, regardless of specialty.

### HIV/AIDS

HIV/AIDS continues to be a public health concern for our country, state, and city (32). In 2023, there were about 39,200 new HIV diagnoses among individuals aged 13 years and older, with a diagnosis rate of 13.7 per 100,000 population (33). Men, especially Black/African American individuals, are disproportionately affected. More than 1.13 million people aged 13 years and older are living with diagnosed HIV in the U.S., and racial disparities persist (33). In Chicago, there were 818 HIV diagnoses reported, an increase of 12.5% compared with 2023, which may indicate a rise in new HIV diagnoses (34). The HIV/AIDS concentration aims to educate future health care professionals about emerging advances in management and new preventative tools.

### Homelessness

Homelessness remains a critical public health issue in our urban area, which is one of the largest cities in the United States, with a population of 2.7 million (8, 35). People experiencing homelessness report multiple health problems, including chronic conditions such as substance use disorder, hypertension, and asthma. Limited access to primary and preventive care often leads them to rely on emergency room care (36). In Chicago, the 2024 Point-in-Time count found 18,836 people to be experiencing homelessness. Black/African American residents continue to be disproportionately affected (37). Evidence suggests that people experiencing homelessness are willing to seek care if deemed important (38–41). Homelessness concentration provides students with the opportunity to learn how the social determinants of health, social injustices, and unmet needs impact this population. In addition, because the course is interprofessional, students experience and learn how various professionals can collaborate to have a greater impact on their care.

### Intimate partner violence (IPV)

IPV is a serious, growing, and preventable public health problem that affects millions of Americans. The harmful impact of IPV resonates across communities, locally and globally. A growing body of evidence highlights the health effects of IPV, including fatal and non-fatal injuries. The consequences of IPV can span a lifetime and include both individual and societal level costs. The cost of IPV against women exceeds an estimated $5.8 billion annually (42–44). These estimates do not include the costs associated with the criminal justice system in IPV matters; thus, the actual cost of IPV is even higher. Evidence also indicates that women carry a disproportionate burden of IPV and suffer more injuries and fatalities than do men (45), and IPV is a significant issue in immigrant families (46, 47). In 2025, IPV incidents made up of 26% Chicago’s violent crime (48), and IPV in Chicago continues to highlight the disparities across racial groups and gender, with marginalized communities experiencing disproportionate risk and barriers to support (49). The IPV concentration enables students to better understand health inequities and the lack of healthcare, particularly given that the absence of a usual source of care and supportive services for this underserved group is a primary care and public health problem.

### Immigrant/refugee health

Chicagoland is home to a rapidly growing and diverse immigrant population. Our large suburban area is home to about 54% percent of the state’s undocumented population, compared to 36% who live in the city (50). The refugee population is concentrated in the urban metropolitan area. Immigrant groups face a host of cultural, linguistic, and other perceived barriers to regular health care. They are less likely to have health insurance and to access health-care resources (51–54). Immigrant and refugee communities in Chicago experience unique health challenges shaped by socioeconomic conditions, language barriers, and differential access to care. This concentration enables our students to develop as a culturally sensitive health professions workforce and trains them in exploring how health systems can be designed to proactively care for and meet the special needs of this underserved population.

### Incarcerated populations

The U.S. incarceration rate has risen by 220% between 1980 and 2014, which can be attributed to state and federal policy changes that have enacted more severe sentencing (55). National data reveal stark disparities. High incarceration rates disproportionately affect racial/ethnic minorities. Among men aged 18 to 64, 1 in 87 white men were incarcerated compared to 1 in 36 Hispanic men and 1 in 12 black men (56). Research indicates that people without high school diplomas or GEDs have a greater likelihood of being incarcerated than their more educated peers (56). These inequities reflect structural barriers in education, employment, and access to social resources, reinforcing cycles of disadvantage that extend beyond confinement and contribute to broader health disparities.

Illinois is among the top 10 states that have the highest prison populations in the country (57). According to the John Howard Association, our state’s prison population has increased by over 500% since 1974 (57). Chicago plays a central role in incarcerating individuals throughout Illinois. Cook County Jail in Chicago is one of the largest single-site jails in the country, with an average daily population of 9,000 and admitting roughly 100,000 detainees (58). Black Chicagoans comprise most of the incarcerated population despite representing one-third of the city’s residents, highlighting the persistent racial inequities (59). Additionally, a large urban Juvenile Temporary Detention Center in Chicago is a medium- to low-security facility that provides temporary secure housing for youth ages 10 to 16 awaiting adjudication (60). The detention center seeks to provide a safe, secure, and caring environment, with programs and structures that enhance youth’s personal development and improve opportunities for success upon return to their communities (60, 61). This course concentration affords our students a unique opportunity to acquire knowledge about this vulnerable population by working with clients at JTDC, an experience typically not offered in health science curricula.

## Pedagogical framework and principles, competencies underlying the educational activity

Interprofessional education (IPE) aims to bring together different professionals to learn with, from, and about one another, enabling them to collaborate more effectively in delivering safe, high-quality care to patients/clients. An emerging body of scholarship supports the value of interprofessional education in preparing the health workforce (62–64). In line with the evidence and national recommendations, we believe that a curriculum structure that supports interprofessional training experiences working directly with underserved populations is essential for health professions training to be collaborative and effective.

Emerging evidence regarding the value of health care delivered via effective interdisciplinary teams has highlighted priorities for interprofessional education (65–67). Globally, the WHO also recommends making interprofessional education and collaborative practice a key priority in the training and practice of health professionals (67). According to the WHO’s *Framework for Action on Interprofessional Education & Collaborative Practice*, IPE is needed to enable current and future health professionals to become part of a “collaborative practice-ready health workforce that is better prepared to respond to local health needs” (67). More details about the relevance of IPE emphasized by the different professions participating in the IAHD course are summarized in the introduction section above on pages 4–5.

Community-based participatory research (CPBR) is a collaborative approach to research that equitably involves all partners, including community members, organizational representatives, and researchers in all aspects of the research process, and recognizes the unique strengths that each brings to the table (68–71). This special approach to research begins with a topic important to the community and aims to combine knowledge with action to achieve social change through research and practical interventions that address the needs of vulnerable populations. As a well-recognized research approach, CBPR offers numerous advantages over traditional research, making it more effective in addressing complex health disparities and achieving social change. By shifting the power dynamic from “researching on” to “researching with” communities, CBPR offers significant advantages over traditional research in addressing complex health disparities and achieving sustainable social change (72).

### Curriculum development approach and background

Utilizing Kern’s Principles for Curriculum Development (73), which include problem identification, needs assessment, determining goals and objectives, identifying educational strategies, implementation, and evaluation and feedback, we developed a two-semester interprofessional course. We utilized CBPR as the primary training modality. This was considered essential by the participating interprofessional faculty team for preparing future health professionals with the skills to effectively address the rising burden of crucial public health concerns and health inequities.

The IAHD Course was developed based on evidence and a careful needs assessment and integrated into a sound foundation of prior curricular framework, the longitudinal Patient-centered Medicine Scholars Program (74). In 2008, we piloted our first IPE learning experience, the Interdisciplinary Service Learning Program, an elective for third-year medical and pharmacy students. The context and rationale for this learning experience were based on the need for interprofessional teamwork as a crucial factor in the effective delivery of healthcare to all patients and communities, particularly those who are vulnerable and underserved (10). Based on participants’ feedback, this pilot educational activity enabled medicine and pharmacy students to learn the value of functioning effectively in interprofessional teams (17). This pilot work was the forerunner to our current IPE-based expanded program.

In 2013, the first author, Dr. Hasnain, received the prestigious Macy Faculty Award from the Josiah Macy Jr. Foundation. This award is presented to select educators nationwide to accelerate necessary reforms in health professions education that accommodate the dramatic changes occurring in medical practice and healthcare delivery. For her Macy Faculty Award project, Dr. Hasnain led the development and implementation of the IAHD course, a two-semester interprofessional course offered during the Fall and Spring semesters for health professions students at UIC.

Working with a team of faculty from participating UIC health science colleges and established community partners, we successfully designed, developed, and launched the new IAHD course in 2014 as the fourth-year component of the longitudinal Patient-centered Medicine (PCM) Scholars Program. Establishing IAHD involved substantial groundwork, including securing the engagement of leadership from various health professions colleges at UIC, assembling a team of interprofessional faculty colleagues to serve as course faculty, developing a comprehensive curriculum, and recruiting students. This process required substantial time and energy, made possible by building on our successful prior work. See Table 1 for a history of program development.

**Table 1:** Program development history.

Year established	Project title/funding	Program/course/students/duration
2005	Training Culturally Responsive Physicians/American Medical Student Association	Service Learning Program (SLP) for 2nd year medical students
2007	A Longitudinal Continuity of Care Predoctoral Curriculum to Promote Patient-centered Medicine/Health Resources and Services Administration, U.S. Department of Health and Human Services	Patient-centered Medicine (PCM) Scholars Program for all levels of medical students
2008	An Interdisciplinary Service Learning Experience to Prepare Tomorrow’s Health Care Professionals/ Association for Prevention Teaching and Research	Interdisciplinary Service Learning Program (ISLP) elective for 3rd year medical and pharmacy students
2014	Longitudinal Team-based Interprofessional Education to Care for Special Needs Populations/Macy Faculty Scholars Award, Josiah Macy Jr. Foundation	Interprofessional Approaches to Health Disparities (IAHD) course for 4th year medical, graduate nursing, 3rd year pharmacy, 4th year dentistry, and graduate public health students

Our programmatic competencies related to interprofessional collaboration were drawn from the competency domains proposed by the Interprofessional Education Collaborative (IPEC) in 2011 (75). The IPEC represents six national education associations of schools of the health professions in the United States: the American Association of Colleges of Nursing, the American Association of Colleges of Osteopathic Medicine, the American Association of Colleges of Pharmacy, the American Dental Education Association, the Association of American Medical Colleges, and the Associated Schools of Public Health. In 2011, IPEC developed a framework for effective collaborative practice and published it as *Core Competencies for Interprofessional Collaborative Practice* (75, 76). Updates to the IPEC Core Competencies were subsequently published in 2016, and in 2023 (76, 77). The 2011 IPEC report highlighted the need for a unifying concept to facilitate the clear development of core competencies for interprofessional collaborative practice (75). This consensus document outlined 38 competencies across four domains: Values/Ethics for Interprofessional Practice (10 competencies), Roles/Responsibilities (9 competencies), Interprofessional Communication (8 competencies), and Teams and Teamwork (11 competencies) (75, 78).

## Methods—learning environment, activities, and evaluation

The IAHD course offers students in medicine, nursing, pharmacy, dentistry, and public health the opportunity to work in teams on community-based participatory research projects. In this mentored interprofessional learning environment, future leaders in the health professions acquire skills to effectively address the rising burden of key public health concerns. The IAHD is an academic year (two-semester) interprofessional course offered during the Fall and Spring semesters for health professions students. The *goal* is to equip students with essential skills to improve healthcare for underserved populations and reduce health disparities through interprofessional education and collaborative practice, utilizing Community-based Participatory Research (CBPR) as a foundational methodology to advance health equity.

### Learning objectives

The IAHD course is designed to enable participating students to:

- Effectively engage in identifying and addressing social determinants of health impacting vulnerable populations;
- Acquire working knowledge and hands-on experience with community-based participatory research (CBPR);
- Develop an interprofessional community-based research project designed to improve health care access, communication, care coordination, or additional priority issues for vulnerable populations;
- Develop skills for functioning as effective members of interprofessional teams; and
- Develop skills for leadership, advocacy, and scholarship.

### Students and selection process

Students in the UIC Colleges of Medicine, Nursing, Pharmacy, Dentistry, and Public Health are invited to apply to the program each summer. The application includes a brief essay, “*Why I want to be an IAHD Scholar*,” and applicants may indicate their preferred vulnerable population: Geriatrics, HIV/AIDS, Homelessness, Immigrant and Refugee Health, Incarcerated Populations, or Intimate Partner Violence. The IAHD course interprofessional faculty team reviews and selects qualified applicants. We make every effort to provide students with their choice of vulnerable population. To ensure an optimal faculty-to-student ratio, the total course enrollment is limited to 30 per year, without a cap on individual professions. See Table 2 for learner levels, program placement within their respective health science college curricula, and credit hours.

**Table 2:** Course participation and credit.

Placement	Medicine	Nursing	Pharmacy	Public health	Dentistry
Student level	M4	Graduate-level students	P3	2nd year MPH students	D4
Place in curriculum	PCM scholars program self-designed elective	Independent study and NUPR 595	Independent Study PMPR 445 & 446	Students confirm with their academic advisor if the course contributes to the IPE requirement	Independent Elective

M4 = medical student year 4; P3 = pharmacy student year 3; D4 = dentistry student year 4. Educational credit is determined by each student’s school or college.

### Faculty selection and involvement

The IAHD course faculty includes a course Director and instructors from the health science colleges representing the students participating in the course. The interprofessional faculty are selected based on their expertise and commitment to the IAHD course’s focus areas, IPE, and CBPR. There are at least two faculty members for each course concentration offered in any given year. Faculty participate on a voluntary basis. The faculty provide ongoing guidance, mentoring, and feedback to students throughout the training. They also actively participate in the development, implementation, and dissemination of the CBPR projects.

### Key learning activities

The IAHD course commences in the Fall Semester with an “Orientation Seminar” that focuses on an overview of health inequities, interprofessional education, and the collaborative process. At the orientation session, scholars meet their interprofessional teams, faculty, and community site representatives to develop a road map for the year. Early on, it is emphasized that there is *no professional hierarchy* among students from different professions. Throughout the course, students work in interprofessional teams and engage in a series of didactic (in-person and online) and experiential learning activities, including CBPR training, to develop essential skills to improve health care for underserved populations. Interprofessional student teams collaborate with staff and clients from community agencies, focusing on those that serve vulnerable populations. Students design CBPR projects around important health issues identified by the community agency and focused on their selected concentration.

The detailed IAHD course syllabus is available through the Curriculum Collection curated by the National Collaborative for Educational to Address Social Determinants of Health (79), which is available for free of cost download.

### CBPR training

Students receive training in CBPR methods during the course. Training sessions are led by interprofessional faculty from the various health professions schools represented in the program. Under the guidance of faculty mentors, interprofessional student teams attend a three-hour monthly seminar and participate in community site visits to develop CBPR projects. Community partners are also invited to the monthly seminars and are encouraged to participate as their time permits. Student teams meet with their faculty leaders and community site partners in weekly team meetings.

### CBPR research

Interprofessional student teams engage in a mentored research process to identify and address priority health needs of vulnerable populations served by our partnering community agencies. The collaborative model employs a team-based approach, working closely with community partners, faculty leaders, and student teams in conducting thorough needs assessments and piloting small changes through Plan-Do-Study-Act (PDSA) cycles (80). The PDSA cycle is a four-step problem-solving model used to improve a process or implement change. The principal focus of CBPR projects is to determine research priorities and intended outcomes in collaboration with the community stakeholders. For example, a desired outcome might be improving access to and retention in high-quality, competent care and services for vulnerable patients who have never received care or have dropped out of care. Community agencies identify priority areas based on their insights and lived experiences, and student teams collaborate to design and pilot-test ideas most likely to succeed and have the greatest impact. The teams create joint ownership of projects, develop research design and methods, including action steps, implement projects, evaluate impacts, and tailor interventions based on findings. CBPR projects, in their truest sense, are determined, co-created, and co-led by community agencies and typically take years to develop. For training purposes in the IAHD course, students may build upon projects from prior years. We make every effort to develop each step of the research process through collaborative decision-making. Student and faculty teams collaborate closely with community partners to design, implement, and evaluate the CBPR projects.

Learning activities are grounded in reflection, self-awareness, collaborative learning, and applied practice, promoting the successful acquisition of core competencies that address the health needs of vulnerable populations. In addition to a carefully curated set of readings and resources that is regularly updated as new evidence becomes available, IAHD scholars utilize a textbook on CBPR (81) and a set of research modules developed specifically for the course. Topics for the research modules include: Major Study Designs; Defining Variables and Selecting Measurement Techniques; Sampling Methods; Measures of Central Tendency; Measures of Variability; Core Concepts in Hypothesis Testing; Measures of Association, Bias and Confounding; Common Statistical Tests; Organizing Data in Tables; Visualizing Data in Graphs; Introduction of Advanced Statistical Techniques; and Statistical Software.

### Reflection and scholarship

Students participate in monthly seminars. Sample seminar topics include: CBPR Methodological and Ethical Considerations; Planning and Conducting CBPR; Analyzing and Interpreting CBPR Findings; Using CBPR to Promote Social Change and Public Health Policy; and Interprofessional Teamwork. In addition to course faculty, we invite guest speakers who are experts on the topic areas covered by the course. Student scholarship includes synthesizing their work through discussions and in writing. Students present their work at the end-of-course concluding Showcase. Upon completion of the coursework, students are encouraged to disseminate their findings at national and local scientific meetings and to share their scholarship through publications.

### Unique aspects of the program

Students have the opportunity to: (1) improve skills for functioning as effective members of interprofessional teams; (2) gain working knowledge and experience of CBPR; (3) improve health care access, communication and care coordination for vulnerable populations; (4) develop expertise in one area of concentration; and (5) apply principles of teamwork and collaborations to address health disparities and inequities.

### Program evaluation

A mixed-methods program evaluation design was used to gather students’ perspectives and experiences in the IAHD course. The goal of the evaluation was to allow participating students to provide both quantitative and qualitative feedback to determine the impact of the learning experiences on the attainment of the course’s learning objectives. Our program evaluation used the first three levels of Kirkpatrick’s Four-Level Training Evaluation Model, which assesses program participants’ reactions, learning, behaviors, and results (82). The program evaluation study was approved by the University of Illinois Institutional Review Board for the Protection of Human Research Subjects, categorized as exempt. Beginning in 2017, students’ acquisition of core competencies for interprofessional collaboration was measured using the IPECC-SET tool (83), developed and published by the first author and colleagues in 2017, and administered in a retrospective pre-post design at the end of the course. The retrospective pre-post design measures students’ learning only at the end by asking them to evaluate what they know from two self-reported viewpoints – before and after participating (84, 85). The responses can be compared to show changes in knowledge or skills. We intentionally selected the retrospective pre-post evaluation design over the traditional pre-post method, as the latter may compromise accuracy when students over- or underestimate their knowledge or ability on the pre-test, given that we often “do not know what we do not know,” hence misrepresenting the true impact of the program (84–86).

Participation in the program evaluation was voluntary, and students were not penalized if they chose not to participate. Students were invited to complete the program evaluation immediately after the course ended.

Data analyses included comparisons of pre-post differences in self-efficacy for competence in interprofessional collaborative practice, measured using the 38-item Interprofessional Education Collaborative Competency Self-Efficacy Tool (IPECC-SET), a validated instrument (1). The pre-post difference in IPECC-SET responses was analyzed using paired-samples t-tests. Descriptive analysis of quantitative data was conducted to explore the extent to which the course learning objectives were met. Thematic analysis of open-ended data was conducted to delineate themes related to the course’s most effective aspects, its influence on students’ abilities to work in interprofessional teams, and how working and learning with other professions broadened students’ perspectives and contributed to their preparation as future health professionals.

## Results

Since 2014, 202 students from UIC’s Colleges of Medicine, Nursing, Pharmacy, Public Health, and Dentistry have participated in IAHD, working in selected focus areas: Geriatrics, HIV/AIDS, Homelessness, Immigrant and Refugee Health, Incarcerated Populations, and Intimate Partner Violence (Table 3). All student teams developed and carried out CBPR projects.

**Table 3:** IAHD course participants by program year.

Program year	Medicine	Nursing	Pharmacy	Public health	Dentistry	Total
2014–2015	13	1	10	2	-	26
2015–2016	16	0	0	2	-	18
2016–2017	12	0	0	0	-	12
2017–2018	9*	5	3	1	-	18
2018–2019	9	6	7	2	-	24
2019–2020	4	5	6	0	3	17
2020–2021	9	4	8	4	3	28
2021–2022	7	2	4	0	0	13
2022–2023	6	2	3	2	2	15
2023–2024	7	3	2	1	1	14
2024–2025	9	7	0	0	1	17
Total	100	35	43	14	10	202

*Includes one pre-med student; dentistry joined in 2019.

Overall, program evaluation findings indicated that students view the learning experience positively. The following section provides the results of quantitative and qualitative data analyses.

### Quantitative findings

- Student-reported agreement with the attainment of course learning objectives has steadily improved over the years as we have refined the course. The cumulative percent agreement for attainment of course objectives ranged from 90.1 to 95.5% (see Table 4).
- Student-reported self-efficacy for interprofessional collaborative practice, measured by the IPECC-SET tool, indicated pre-post differences in a positive direction. Statistically significant (*p* < 0.001) increases in self-efficacy for competence in interprofessional collaborative practice were observed across all 38 items of the IPECC-SET, in all four domains. The range of mean changes was 0.4–1.9. The largest increases (means >1.5) were observed in six items across the four domains: two in Values and Ethics (VE), one in Roles and Responsibilities (RR), one in Interprofessional Communication (CC), and two in Teams and Teamwork (TT) (see Table 5).

- VE 5. Work with others who contribute/support delivery of prevention & health services (mean 2.0, SD 1.8; *p* < 0.0001)
- VE 8 Manage ethical dilemmas (mean 1.8, SD 1.9; *p* < 0.0001)
- RR 7. Forge interdependent relationships with other professions (mean 1.5, SD 1.5; *p* < 0.0001)
- CC 8. Communicate consistently the importance of teamwork in patient-centered and community-focused care (mean 1.8, SD 1.6; *p* < 0.0001)
- TT 3. Engage other health professionals—appropriate to the specific care situation—in shared patient-centered problem solving (mean 1.8, SD 1.8; *p* < 0.0001)
- TT 9. Use process improvement strategies to increase teamwork (mean 1.7, SD 1.5; *p* < 0.0001)

**Table 4:** Student-reported attainment of learning objectives, 2014–2025, *N* = 132.

The IAHD course enabled students to	Agree (%)	Neutral (%)	Disagree (%)
Effectively engage in identifying and addressing social determinants of health impacting vulnerable populations	126 (95.5)	1 (0.7)	5 (3.8)
Acquire working knowledge and hands-on experience with community-based participatory research and quality improvement methods	126 (95.5)	1 (0.7)	5 (3.8)
Develop an interprofessional community-based research project designed to address health disparities	125 (94.7)	3 (2.3)	4 (3.0)
Develop skills for functioning as effective members of interprofessional teams	119 (90.1)	3 (2.3)	10 (7.6)
Develop skills for leadership	122 (92.4)	4 (3.0)	6 (4.5)
Develop skills for advocacy	120 (90.9)	4 (3.0)	8 (6.1)
Develop skills for scholarship	120 (91.6)	5 (3.8)	6 (4.6)

**Table 5:** Differences in pre- post- mean IPECC-SET scores 2017–2025, *N* = 106.

Variables	Pre-mean (SD)	Post-mean (SD)	Mean^a^ (SD)	*p*-value**
Values and ethics for interprofessional practice				
VE 1. Place patient’s interests at the center of interprofessional health care delivery	7.1 (1.9)	8.3 (1.8)	1.2 (1.4)	<0.0001
VE 2. Respect dignity and privacy of patients	7.8 (1.6)	8.4 (1.3)	0.6 (0.8)	<0.0001
VE 3. Embrace cultural diversity and individual differences of patients, populations and team members	7.8 (1.4)	8.4 (1.0)	0.6 (0.9)	<0.0001
VE 4. Respect cultures, values, roles, and expertise of one another	7.7 (1.5)	8.4 (1.1)	0.7 (1.1)	<0.0001
VE 5. Work with others who contribute/support delivery of prevention & health services	5.9 (2.1)	7.9 (1.4)	*2.0 (1.8)	<0.0001
VE 6. Develop a trusting relationship with patients, families and team membersSS	7.4 (1.6)	8.2 (1.2)	0.8 (0.9)	<0.0001
VE 7. Demonstrate high standards of ethical conduct	7.5 (1.6)	8.2 (1.2)	0.7 (1.0)	<0.0001
VE 8. Manage ethical dilemmas	5.6 (2.1)	7.4 (1.8)	*1.8 (1.9)	<0.0001
VE 9. Act with honesty and integrity in relationships with patients, families and other team members	8.1 (1.4)	8.5 (0.9)	0.4 (0.7)	<0.0001
VE 10. Maintain competence in own profession appropriate to scope of practice	7.4 (1.6)	8.2 (1.2)	0.8 (1.1)	<0.0001
Roles & responsibilities				
RR 1. Communicate roles and responsibilities to patients, families, and team members	7.5 (1.5)	8.2 (1.1)	0.7 (1.0)	<0.0001
RR 2. Recognize one’s limitations in skills, knowledge, and abilities	7.0 (1.8)	8.0 (1.3)	1.0 (1.2)	<0.0001
RR 3. Engage diverse healthcare professionals who complement one’s professional expertise to develop strategies to meet patient care needs	7.0 (1.8)	8.1 (1.3)	1.1 (1.3)	<0.0001
RR 4. Explain roles and responsibilities of care providers and how the team works to provide care	6.9 (1.9)	8.0 (1.6)	1.2 (1.4)	<0.0001
RR 5. Use the full scope of knowledge, skills and abilities to provide care that is efficient, timely, effective, safe and equitable	7.1 (1.8)	8.0 (1.3)	0.9 (1.2)	<0.0001
RR 6. Communicate with team members to clarify responsibilities in executing treatment plan	6.9 (1.6)	7.9 (1.5)	1.0 (1.3)	<0.0001
RR 7. Forge interdependent relationships with other professions	6.5 (1.9)	8.0 (1.5)	*1.5 (1.5)	<0.0001
RR 8. Engage in continuous professional and interprofessional development to enhance performance	6.7 (1.8)	8.1 (1.1)	1.4 (1.6)	<0.0001
RR 9. Use unique and complementary abilities of all members to optimize patient care	6.7 (1.9)	8.0 (1.1)	1.3 (1.4)	<0.0001
Interprofessional communication				
CC 1. Choose effective communication tools and techniques	6.8 (2.0)	7.9 (1.4)	1.1 (1.2)	<0.0001
CC 2. Organize and communicate information with patients, families, and team members that is understandable	6.9 (1.8)	7.9 (1.5)	1.0 (1.0)	<0.0001
CC 3. Express knowledge and opinions to team with confidence	6.6 (1.8)	8.0 (1.3)	1.4 (1.3)	<0.0001
CC 4. Listen actively and encourage ideas of team members	7.4 (1.8)	8.3 (1.2)	0.9 (1.4)	<0.0001
CC 5. Give timely, sensitive, instructive feedback to others about their performance responding respectfully as a team member to feedback from others	6.5 (2.1)	7.5 (1.6)	1.0 (1.4)	<0.0001
CC 6. Use respectful language appropriate for difficult situations or conflicts	7.4 (1.7)	8.2 (1.2)	0.8 (1.1)	<0.0001
CC 7. Recognize one’s uniqueness contributes to effective communication, conflict resolution and positive working relationships	6.8 (1.8)	8.1 (1.3)	1.3 (1.5)	<0.0001
CC 8. Communicate consistently the importance of teamwork in patient-centered and community-focused care	6.3 (2.1)	8.1 (1.3)	*1.8 (1.6)	<0.0001
Teams and teamwork				
TT 1. Describe process of team development and the roles and practices of effective teams	6.3 (1.9)	7.7 (1.5)	1.4 (1.4)	<0.0001
TT 2. Develop consensus on ethical principles to guide patient care and teamwork	6.8 (1.9)	7.8 (1.6)	1.0 (1.3)	<0.0001
TT 3. Engage other health professionals—appropriate to the specific care situation—in shared patient-centered problem solving	6.0 (2.3)	7.8 (1.3)	*1.8 (1.8)	<0.0001
TT 4. Integrate knowledge & experience of other professions—appropriate to the specific care situation—to inform care decisions while respecting patient and community values and priorities/preferences for care	6.8 (1.7)	8.0 (1.3)	1.2 (1.4)	<0.0001
TT 5. Apply leadership practices that support collaborative practices and team effectiveness	6.9 (1.8)	8.0 (1.4)	1.1 (1.3)	<0.0001
TT 6. Engage self and others to constructively manage disagreements about values, roles, goals and actions	6.54 (2.0)	7.5 (1.7)	1.0 (1.3)	<0.0001
TT 7. Share accountability with other professions, patients and communities for outcomes relevant to prevention & healthcare	6.9 (1.8)	8.1 (1.3)	1.2 (1.5)	<0.0001
TT 8. Reflect on individual & team performance for improvement	6.8 (1.8)	7.9 (1.5)	1.1 (1.5)	<0.0001
TT 9. Use process improvement strategies to increase teamwork	5.6 (2.3)	7.3 (1.9)	*1.7 (1.5)	<0.0001
TT 10. Use available evidence to inform effective teamwork and team-based practices	6.9 (1.9)	7.9 (1.5)	1.0 (1.3)	<0.0001
TT 11. Perform effectively on teams in a variety of settings	6.8 (1.7)	8.1 (1.3)	1.3 (1.3)	<0.0001

a Mean difference, pre- and post- paired t-tests.

*Pre- and post- mean difference >1.5. **p-value < 0.05.

### Qualitative findings

Students’ responses to three key open-ended program evaluation questions are summarized below.

#### Most effective aspects of the program

Analysis of students’ responses yielded two major themes regarding the aspects of the course that were considered most effective:

##### Interprofessional teamwork

Selected quotes:

“*The interprofessional team was incredibly effective because it helped to open my mind and give me other perspectives towards health care. I also really enjoyed being able to create our own project. Having ownership over the project gave me insight into the challenges of community-based participatory research and how to overcome those challenges.”*

*“The most effective part of IAHD were Interaction with other professionals from different fields, collaboration with a community organization, guest lecturers.”*

##### Community-based work

Selected quotes:

*“The community partner and patients. The most valuable aspect of this program is the community work. Working in the communities and with the people.”*

*“The IAHD program provided access to essential shelters and resources, emphasized practical training equipping participants with the skills required to address real-world health challenges and bridging the gaps in healthcare access and quality for those in need.”*

#### Most important contributions team members made to students’ learning

Analysis of comments highlighted how working and learning with other professions yielded one broad theme:

#### Broadened the students’ perspectives and contributed to their preparation as future health professionals

Selected quotes:

“*My team members mostly helped me open my mind. I looked at a lot of things through the eyes of a medical student, and that caused some slight tunnel vision at times. Having other students with other backgrounds made sure that I did not ignore certain aspects of the project and how to help our patients.*”

*“I really enjoyed getting to know my team members and see how our knowledge differs greatly. We were each able to contribute and learn from each other. We were great at communicating and that showed me the promise in this kind of work in the future. On the other hand, working with six very different schedules (not including the faculty and community partner) showed me the difficulty and time-consuming nature of this type of work. I think it is good to see it in an academic setting to better prepare for the future.”*

#### Influence on students’ future work as future health professionals

Analysis of students’ comments to this question yielded three major themes:

##### Enhanced understanding of patient, community perspectives, and social determinants of health

Selected quotes:

*“I have a better appreciation for the challenges that some of my patients will face when they are discharged from the hospital. I have a better appreciation for the outside factors that affect health.”*

*“I will be extremely mindful to consider my patient's situation or going into a community with the idea to "fix" what's wrong. Rather, I will try to understand where they come from and how to relate based on their situation.”*

##### Enhanced ability to work with other professions

Selected quotes:

*“I have a much better understanding of my role in a team like this now. I now understand how social work, pharmacy, and public health professionals can all help to improve outcomes for patients. I am much more likely to participate in a team like this in the future based on my participation in this project.”*

*“As a member of an underserved community, I have first-hand knowledge of the inadequate healthcare opportunities that are available to underserved populations. Programs like IAHD allow students to learn that the healthcare system can only be fixed by working together. Doctors, nurses, pharmacists, social workers, and public health professionals all have something to offer to underserved communities. I plan to seek out interprofessional opportunities in my practice to continue help decrease healthcare disparities in underserved communities.”*

##### Enhanced ability to provide patient-centered care

Selected quotes:

*“I think the main thing I will do now is listen. Listening to the people facing disparity is the most important thing, and just giving a title to it doesn't do it justice. No one but the people experiencing inequity can speak to their experience, and I plan on focusing a lot more on listening to these people to tailor my efforts to them.”*

*“It will help me communicate better with my patients as I feel more comfortable with the topic. I think these are topics that often do not get asked about enough since they are sensitive, but my interactions with the individuals showed me that they wanted people to ask about and reach out sooner.”*

A list of students’ CBPR Projects by course year is presented in the Appendix.

## Discussion

This paper describes the development and initial outcomes of an initiative to equip students in medicine, nursing, pharmacy, dentistry, and public health with essential skills to improve healthcare for underserved populations and address health disparities through interprofessional education and community-based participatory research, grounded in campus-community partnerships. Based on students’ program evaluation feedback, the program is making meaningful contributions to their preparation as future health professionals.

Providing patient-centered care is one of the cornerstones of quality, as designated by the IOM, which also highlights a “quality chasm” between the healthcare currently existing in the U.S. and the healthcare patients *could* have (67, 75, 76). To help bridge this quality chasm, the University College of Medicine initially developed and implemented a longitudinal curriculum in patient-centered medicine, known as the PCM Scholars Program, which later evolved to include an interprofessional training course, the IAHD. This work aligns with the frameworks proposed by the IPEC and the World Health Organization (WHO) (9–16, 77).

As medicine and health care change, the standard question “How can we improve quality and lower cost?” is being reformulated to “Can we improve quality of care and lower medical costs by ensuring that the neediest patients receive equitable care?” For health professions educators, this question becomes even more crucial, as the current system of health professions education does not provide a cohesive structure for training health science students to provide care to underserved populations.

There are numerous calls to transform health professions education by the professions of medicine, nursing, pharmacy, dentistry, and public health—the professions participating in our educational innovation—in order to (1) prepare the future health professions workforce to meet the public’s needs and expectations, (2) provide more training in population health and the role social factors play in effecting health change, (3) community-based settings be used more and hospital settings less as training sites, (4) traditional models of training health science students evolve to provide more interprofessional and team-based learning (68–71).

The IAHD course provides a team-based training program for students in medicine, nursing, pharmacy, dentistry, and public health at the University of Illinois Chicago. This interprofessional training program responds to the national call for health professions education to prepare our future health workforce to face the 21st-century challenge to address health disparities by training them in CBPR methods (75–77), equipping them with the tools they need to improve health care delivery.

Our programmatic competencies related to interprofessional collaboration encompass the domains of values and ethics, roles and responsibilities, interprofessional communication, and teams and teamwork as defined by the IPEC Core Competencies (67), as well as the WHO’s *Framework for Action on Interprofessional Education & Collaborative Practice* (83), which highlights that IPE is needed to enable current and future healthcare professionals to become part of a “collaborative practice-ready health workforce that is better prepared to respond to community health needs.” Our use of a validated tool (86) to measure self-efficacy for interprofessional collaborative practice was a key strength of our program evaluation design. The findings showing post-course increases in self-efficacy for interprofessional collaborative practice highlight the value of creating educational interventions that enable interprofessional student teams to work together and learn from one another through community-engaged experiential learning.

Health inequities in our urban settings are real and well-documented (87, 88). Although addressing health inequities has garnered significant attention, the literature suggests that training in this area is still in its early stages of development, and much remains to be done (3, 88). Although IPE can be viewed as curriculum (what material is learned) or an instructional method (how material is learned), its real promise lies in its role as a lever for promoting change (89). A key aspect of our work involves developing rigorous tools to evaluate the effects of interprofessional education and collaborative practice (83, 90). Furthermore, creating a shared taxonomy among the health professions can serve to streamline and synergize educational activities and related assessment and evaluation efforts.

### Limitations

The findings from our effort to create transformative interprofessional education to prepare future health professionals are promising, but our work also has certain limitations.

1. The course may have limited generalizability as it is implemented in a single setting. Other educational settings can adapt our work according to their situational needs.
2. Our evaluation findings are primarily drawn from student feedback. Although we moved beyond Level 1 in Kirkpatrick’s evaluation framework (73), we still need to examine higher-level changes, which require long-term follow-up. Additionally, expanding the evaluation framework beyond student outcomes to include perspectives from community partners and the clients they serve, as well as faculty perspectives on course implementation and sustainability, will further strengthen our work.

### Lessons learned and future directions

Our work in training health science students utilizing interprofessional education and community-based participatory research, focusing on social determinants of health in focused areas of vulnerability, is making meaningful contributions to advancing the health professions education science towards discovering new models for training the future healthcare workforce to address the needs of our evolving patient populations, reduce health disparities, and advance health equity.

Developing and implementing educational innovations always entails unique challenges. Given the experiential nature of the IAHD course, its training, development, implementation, and evaluation posed multiple challenges. The COVID-19 Pandemic also created special challenges for the IAHD course. For example, we had to pivot and change much of the in-person learning to virtual formats. Fortunately, despite significant hurdles, the course continued without compromising on educational quality while maintaining team safety. The student evaluations, number of students participating, and number of community organizations participating did not change significantly after the shift. The program evaluation findings are carefully reviewed annually by the project team to inform refinements for future iterations.

We have learned several lessons through our journey of program development and implementation. Below are some *key lessons* we continue to pay heed to as we proceed.

#### Curricular transformation

- Curriculum development should be responsive to local, regional, and national trends and emerging needs in health professions education and health care delivery and practice.
- Theory and evidence need to be integrated in curriculum transformation efforts. We have drawn heavily from theoretical and conceptual frameworks, including Kern’s principles of curriculum development (73) Kotter’s principles of change management (91), IPEC Core Competencies for Interprofessional Collaborative Practice (68–71), CBPR (82, 83), and program evaluation principles. We use these as conceptual frameworks and a core foundation for developing, implementing, and evaluating IPE curricular initiatives at our institution.
- Scholarly work and data-driven processes must be central to program development and continuous quality improvement and should be a key priority. Our mixed-methods program evaluation approach, using validated evaluation frameworks and instruments (88) to measure impact, has strengthened our work.

#### Student and faculty engagement

- Designing and implementing interprofessional education is not an easy task. Student input is essential for developing learner-centered, meaningful educational programs. The “interprofessional aspect” of any IPE program encompasses both faculty and students.
- Recruitment and development of an interprofessional faculty group improve the likelihood of program sustainability and serve as an impetus for other collaborative IPE projects.
- Proactive recruitment and student and faculty development efforts, as well as seeking and incorporating student and faculty feedback in program refinement and continuous quality improvement, are necessary to ensure optimal engagement in interprofessional activities.

#### Institutional challenges

- Institutional support is crucial in providing the necessary infrastructure and support for program development, growth, and sustainability, particularly after the end of extramural funding.
- When people start working individually and in silos, there is a lot of wasted energy and resources. Hence, it is crucial to engage people early on and work together collaboratively.
- Do not be paralyzed by perfection. There are certainly aspects that could be improved in the IAHD course, for example, uniform participation from all participating professions. The course is designed to be interprofessional, but medical students participate in greater numbers compared to other groups. This was initially a great concern for the team; however, with advice from the Macy Foundation mentors, we realized that the lack of such uniformity should not impede curricular deployment. The greater participation in medicine likely stems from the course being part of a larger medical student program. Moving forward, we plan to continue our recruitment efforts to attract more students from diverse professions.

#### Building and sustaining community partnerships and IPE teams

- Successful teams thrive when there is trust, respect, and shared goals and values. Building and sustaining campus-community partnerships is an iterative and challenging process that requires mutual trust, respect, and ongoing nurturing. We learned the key value of intentionally engaging community partners throughout all phases of program development and implementation.
- Effective campus–community collaboration and partnership require more than periodic outreach. It is essential to engage all key partners early, be consistent, and ensure that the activities are mutually beneficial and grounded in the real needs and priorities of the communities. By identifying mutual goals, sharing decision-making, and maintaining open communication, we strengthen the trust and enhance the relevance and impact of our work. These relationships must be nurtured to build sustainability that will endure. Our long-standing community partnerships are a testament to mutual respect and value.
- Focusing on the purpose, meaning, and value of what we set out to do keeps us motivated and helps our teams persevere in the face of challenges. Developing anything that is complex and multilayered can be both exhilarating and exasperating. This is certainly true of curriculum development and charting into new territory, such as interprofessional education and campus-community partnerships. Remembering what a great privilege it is to positively influence the learning of our future health professionals and the health of our communities is an ongoing extrinsic motivator for our team.
- In addition to program development and deployment, it is critical to take time to pause, reflect, and celebrate our successes and learn from any mistakes. Our IPE team has been successful in collaborative program development, scholarship (including papers and presentations), obtaining grant funding, and developing a network of mutually supportive colleagues and friends.

As part of our next steps, we plan to conduct an in-depth study of the community partners’ perspectives on refining the IAHD course and strengthening our Campus-Community partnership. Our longer-term goal is to broaden the interprofessional competencies to better achieve the Quintuple Aim: improving patient experience of care, enhancing population health, reducing the per capita cost of healthcare, and enhancing care team well-being (92), and advancing health equity (93).

### Institutional recognition

The IAHD course received institutional recognition as the winner of the 2021 University of Illinois Inaugural “Interprofessional Teaching in Action Matters” Award (I-TEAM Award) – This award recognizes a faculty team who has demonstrated excellence in interprofessional practice and education (IPE) through teaching innovation and the advancement of the following institutional mission: “*Create transformational change in health professions education and health care service delivery by delivering evidence-based learning experiences that build collaborative competence and foster interprofessional scholarship and collaborative practice across academic programs, clinical services and community partners with focused attention to the pressing needs of underserved individuals and populations*.”

## Conclusion

Given the importance of interprofessional teamwork and the growing need to address the health inequities faced by underserved populations, integrating interprofessional learning experiences into health professions training is highly relevant, feasible, and critically needed. We remain optimistic that our work will lead to meaningful contributions to advancing the science of health professions education and discovering new, collaborative models for training the future health professions workforce to be better prepared to advance health equity. This work will also enable better assessment and addressing the needs of our evolving patient populations through engagement with both a broad interprofessional team and community groups, ultimately reducing health disparities and advancing health equity.

Our work is grounded in a moral compass that calls for social accountability (70, 71) from academic health centers and the expansion of training for our future health workforce to heighten health professions students’ personal commitment to service, social justice, and civic responsibility in addressing historic health disparities and advancing health equity.

*“If we acknowledge the growing body of evidence that healthcare delivered by well-functioning teams produces better results, there is a serious disconnect with the educational system that is still structured in silos.”—*George Thibault, MD, Past President, Josiah Macy Jr. Foundation

## Data Availability

The raw data supporting the findings of this article will be made available by the authors upon request, without undue reservation.
