# Supplementary material for: Transformative interprofessional education and campus community partnerships to prepare the health professions workforce for advancing health equity
Source: Front Public Health. 2026 Mar 25;14:1763427. doi: 10.3389/fpubh.2026.1763427 (PMC13057566; doi:10.3389/fpubh.2026.1763427)
Supplement: Supplementary file 1 [file Data_Sheet_1.docx]

**Appendix. IAHD CBPR Projects by Concentration and Year**

| **Concentration** | **CBPR Projects: 2014-2020** |
| --- | --- |
| **Geriatrics** | *2014 – 2015*: Assessment of a Navigation Tool to Assist in Improving Healthcare Activation amongst Senior Residents: A Prospective Study Using Community-Based Participatory Research  *2016 – 2017*: Using Active Remote Care Technology to Enhance Health and Well-being of Home-residing Older Adults: Evaluation of Initial Impacts and Future Directions  *2017 – 2018*: Geriatric Health Literacy: Piloting a Healthcare Appointment Workbook  *2018 – 2019*: Efficacy of Different Curricular Methods of Chronic Disease Self-Management Education Programs Among Community-Dwelling Older Adults  *2019 – 2020*: Enhancing Socialization Through the Arts: Using Art to Address Social Isolation Among Community-Dwelling Older Adults Through Community Engagement and Interprofessional Collaboration  *2020 – 2021:* Mitigating Social Isolation and Enhancing Social Engagement Among Older Adults during the COVID-19 Pandemic through Technology  *2021 – 2022:* Enhancing Community-Dwelling Older Adults’ Self-Confidence to Manage their Health and Well-Being Utilizing the 4Ms Model  *2022 – 2023*: Implementation of Social Activities to Mitigate Loneliness and Improve Mental Health Among Residents at H.O.M.E.  *2023 – 2024*: Utilizing the 4Ms Framework to Increase the Self-Efficacy of Older Adults in Managing their Health |
| **HIV/AIDS** | *2014 – 2015*: Improving Health Literacy at Project VIDA  *2015 – 2016*: HIV/AIDS  *2016 – 2017*: Peer Video Testimonials to Increase Use of PrEP by African American and Latino Communities in Chicago  *2017 – 2018*: PrEP Continuum of Care: Analysis of Outreach to PrEP Initiation and Follow-up  *2018 – 2019*: Increasing PrEP Adherence  *2021 – 2022:* Deficiencies in PrEP Adherence among At-Risk Populations  *2022 – 2023:* Identifying Barriers to Accessing Pre-Exposure Prophylaxis among Latino/x Gay, Bisexual, other MSM, and Trans-Women in Chicago  *2023 – 2024*: Clinical Perspectives Regarding Barriers and Facilitators to PrEP Uptake and HIV Care Among Latine MSM and Transgender Women |
| **Homelessness** | *2014 – 2015*: An Interprofessional Approach to Improving Health Disparities in a Homeless Population  *2015 – 2016*: Nutrition issues for Homelessness  *2016 – 2017*: Homelessness in Chicago  *2017 – 2018*: Nutrition Education & Cookbook Design at the Lincoln Park Community Shelter: A Community Based Participatory Research (CBPR) Pilot Study  *2018 – 2019*: Homelessness and Diabetes  *2019 -2020*: Impact of Naloxone Training on the Homeless Community  *2020 – 2021*: Impact of COVID-19 Education Among People Experiencing Homelessness  *2021 – 2022:* Impact of Stress and Anger Management on Individuals Experiencing Homelessness  *2022 – 2023*: Guided Mindfulness Exercises and Art in a Homeless Population  *2023 – 2024*: Impact of Medical, Dental & Pharmacy Education Sessions at the Lincoln Park Community Shelter  *2024 – 2025*: Health and Wellness Workshops for Chicago Homeless Shelters |
| **Immigrant & Refugee Health** | *2014 – 2015*: Assessing Barriers to Health Care Access amongst Refugees Recently Resettled in Chicago  *2015 – 2016*: Immigrant & Refugee Health  *2017 – 2018*: Preventative Health Care Among Syrian Refugees: Beliefs, Practices, and Experiences  *2018 – 2019*: Employing a Health Fair to Determine the Healthcare Needs of the Syrian Refugee Population  *2019 – 2020*: A Guide Towards a Healthier Lifestyle: Addressing Nutrition, Exercise & Screen-Time in the Syrian Refugee Population  *2020 – 2021*: Mitigating the Effects of COVID-19 Shelter in Place Orders on Syrian Refugee Children’s Health |
| **Incarcerated Populations** | *2019 – 2020*: Lessons Learned: Interprofessional Health Literacy Programming at a Juvenile Temporary Detention Center  *2020 – 2021*: Adaptation of the Preventing HIV/AIDS Among Teens (PHAT) Life curriculum to a virtual format for delivery at the Juvenile Temporary Detention Center  *2021 – 2022:* Sexual and Reproductive Health Care Clinic Mapping Intervention for Justice-Involved Youth  *2022 – 2023*: Improving Health Literacy on Sexually Transmitted Infections and Consent in Male Detained Juveniles  *2023 – 2024*: Improving Health Literacy on Sexually Transmitted Infections and Consent in Male Detained Juveniles  *2024 – 2025*: Improving Sexual Health Literacy in Justice-Involved Youth |
| **Intimate Partner Violence** | *2014 – 2015*: Self-Care Tools and the Effect on Quality of Life for Survivors of Domestic Violence  *2015 – 2016*: Domestic Violence  *2016 – 2017*: Health Care Literacy & Empowerment for Survivors of Domestic Violence  *2017 – 2018*: Rediscovering Her Power: Assessing & Increasing Empowerment Amongst Survivors of Domestic Violence  *2018 – 2019*: Finding My Voice: Navigating the Healthcare System Curriculum  *2019 – 2020*: Enhancing Self-Efficacy and Engagement of Survivors of Intimate Partner Violence (IPV) Regarding Women’s Health  *2020 – 2021*: Impact of Self-Efficacy Health Education for Survivors of Intimate Partner Violence  *2021 – 2022:* Impact of Education Related to Stress Reduction, Childhood Trauma & Childhood Resilience on Survivors of Intimate Partner Violence  *2022 – 2023*: Empowering IPV Survivors in their Path to Healing  *2023 – 2024*: Strengthening the Bond Between Survivors of IPV and Their Children  *2024 – 2025*: Supporting Wellness Among Survivors of Intimate Partner Violence |
